# Clonal Lineages and Virulence Factors of Carbapenem Resistant *E. coli* in Alameda County, California, 2017–2019

**DOI:** 10.3390/antibiotics11121794

**Published:** 2022-12-10

**Authors:** Samuel Slown, Nikolina Walas, Heather K. Amato, Tyler Lloyd, Vici Varghese, Monica Bender, Mark Pandori, Jay Graham

**Affiliations:** 1 School of Public Health, University of California, Berkeley, CA 94704, USA; 2 Alameda County Public Health Laboratory, Oakland, CA 94605, USA

**Keywords:** antibiotic resistance, *E. Coli*, carbapenem, genomic analysis

## Abstract

The prevalence of carbapenem-resistant *Enterobacterales* (CRE) has been increasing since the year 2000 and is considered a serious public health threat according to the Centers for Disease Control and Prevention. Limited studies have genotyped Carbapenem-resistant *Escherichia coli* using whole genome sequencing to characterize the most common lineages and resistance and virulence genes. The aim of this study was to characterize sequence data from carbapenem-resistant *E. coli* isolates (*n* = 82) collected longitudinally by the Alameda County Public Health Laboratory (ACPHL) between 2017 and 2019. *E. coli* genomes were screened for antibiotic resistance genes (ARGs) and extraintestinal pathogenic *E. coli* virulence factor genes (VFGs). The carbapenem-resistant *E. coli* lineages were diverse, with 24 distinct sequence types (STs) represented, including clinically important STs: ST131, ST69, ST95, and ST73. All Ambler classes of Carbapenemases were present, with NDM-5 being most the frequently detected. Nearly all isolates (90%) contained genes encoding resistance to third-generation cephalosporins; *bla*_CTX-M_ genes were most common. The number of virulence genes present within pandemic STs was significantly higher than the number in non-pandemic lineages (*p* = 0.035). Virulence genes *fim*A (92%), *tra*t (71%), *kps*M (54%), and *iut*A (46%) were the most prevalent within the isolates. Considering the public health risk associated with CRE, these data enhance our understanding of the diversity of clinically important *E. coli* that are circulating in Alameda County, California.

## 1. Introduction

Infections caused by carbapenem-resistant *Enterobacterales* (CRE) are a growing public health concern, as carbapenemase-producing organisms have become more prevalent in the human population [1,2]. Carbapenems have exceptional stability against most β-lactamases, have reduced vulnerability to β-lactam resistance determinants, and became the drug of choice for gram-negative bacteria resistant to cephalosporins [3]. Resistance to carbapenems among *Enterobacterales* is mediated by the loss of outer membrane porins, the overexpression of multi-drug efflux pumps, and enzyme-mediated resistance. Enzyme-mediated resistance is of critical clinical importance because these genes can be horizontally transferred and are associated with other clinically relevant resistance genes [4]. Over the past two decades, carbapenem resistance has increased to such a degree that in 2017, the World Health Organization (WHO) released a global priority list of pathogens and ranked CRE in the highest priority category, critical, representing the greatest threat to human health [5].

*E. coli* is associated with significant morbidity and mortality in infected individuals, and an increasing number of clinical reports describe extraintestinal and invasive infections due to these organisms [6]. Pathogenic *E. coli* are separated into pathotypes based upon the diseases they cause, the virulence factors present, and host reservoirs [7]. Extraintestinal pathogenic *E. coli* (ExPEC) are a broad collection of pathotypes that are facultative pathogens colonizing the extraintestinal environment of hosts. ExPECs are the most common gram-negative bacterial pathogens in humans, accounting for the largest percentage of cases of urinary tract infections (UTI’s), sepsis, and bacteremia, with a globally increasing mortality and morbidity [8,9]. ExPEC pathotypes include uropathogenic *E. coli* (UPEC), neonatal meningitis *E. coli* (NMEC), sepsis *E. coli* (SEPEC), and a non-human pathotype avian *E. coli* (APEC) [10]. ExPECs are derived from a small number of phylogenetic lineages, many of which are considered pandemic lineages. Four sequence types (STs), defined using multi-locus sequence typing, are responsible for nearly half of all the *E. coli* urinary tract infections and blood stream infections in the world: pandemic lineages ST131, ST69, ST 95, and ST73 [11]. However, recent studies have also found that ST405 and ST10 are rapidly increasing in prevalence and distribution [12]. Prior to the year 2000, ExPECs were mostly susceptible to first line antibiotics [13]. However, as the use of β-lactam antibiotics to treat ExPEC has increased, highly resistant ExPECs (e.g., ESBL-producing ExPECs) are now widespread and important causes of UTIs and bacteremia [14]. Carbapenem resistance in ExPECs gives rise to clinical treatment challenges, as these bacteria are more likely to be resistant to all β-lactam antibiotics. By combining ESBL antibiotic resistance genes (ARGs) such as *bla*_TEM_, *bla*_SHV_, and *bla*_CTX_, and AmpC β-lactamases such *bla*_CMY_ with carbapenem resistance genes, the antibiotics used to treat these infections becomes limited due to such extensive bacterial resistances [15].

*E. coli* are often resistant to carbapenems due to the production of carbapenemases, a type of β-lactamase enzyme that enables the bacterium to inactivate most β-lactam drugs, including carbapenems. Carbapenemases are separated into molecular classes of β-lactamases: the A, B, C, and D β-lactamases of the Ambler classification. Gram-negative bacteria, such as ExPECs, can disseminate ARGs between members of *Enterobacterales* through mobile genetic elements, acting as vehicles for transferring resistance mechanisms [16]. This horizontal spread of ARGs has resulted in more resistant strains of ExPECS. Much of the current literature concerning CRE focuses on *Klebsiella pneumoniae* due to the pandemic success of KPC-producing *K. pneumoniae* [17], and the dominance of all classes of carbapenemase genes within the genus [18]. There is limited research that characterizes the distribution of specific ARGs, virulence factors, and the correlation between the two within carbapenem-resistant *E. coli*. Although ARGs are a key survival mechanism for infectious ExPECs, the high number of virulence factors contained within ExPECs are attributed to the increased pathogenicity of the organisms [19].

In *E. coli*, the spread of virulence factor genes (VFGs) occurs through horizontal gene transfer [20]. VFGs encode proteins, often found on mobile genetic elements, which contribute to the pathogenicity of ExPECs by enhancing the survival of the bacteria outside the intestines and evading host mechanisms [20]. Although horizontal gene transfer is difficult to detect, we see evidence of its success in ExPECs becoming dominant pathotypes through clonal propagation [21,22]. Within the ExPEC pathotypes there is a large collection of highly conserved VFGs which are commonly divided into subgroups: (1) Adhesins, which enable attachment to host cells, commonly through fimbriae mediated attachment, such as *fim*, *sfa*, and *pap* virulence factors; (2) iron uptake/siderophores, which enable iron sequestration in low iron environments, such as *Iut*A; (3) protectins such as *Kps*M, which prevents phagocytosis or *ompA*, which encodes for a porin, an outer membrane protein; (4) toxins, such as *hyl*D, which creates pores in the host cells, causing cell lysis; and (5) invasins, which aid in the crossing of the blood–brain barrier, such as *ibeB* [10]. The presence of these VFGs is often used as a classification mechanism to identify ExPECs, though there is little consensus as to which VFGs should be used for ExPEC classification [8].

Whole genome sequencing (WGS) has supported a more comprehensive characterization of antibiotic resistant bacteria and their mechanisms. In this study, we characterize and compare sequence data from carbapenem-resistant *E. coli* isolates collected by the Alameda County Public Health Laboratory (ACPHL) in California between 2017 and 2019. As antibiotic resistance is a threat to public health, the ACPHL performs continued surveillance of these critical organisms through a health officer order mandating the submission of carbapenem resistant *E. coli*. As a result of this surveillance, this analysis aimed to evaluate the genomic features of clonal species to enhance the understanding of clinically important carbapenem-resistant *E. coli* circulating in Alameda County, California.

## 2. Results

### 2.1. Characteristics of Clustering, Sequence Types, and Subtypes

Phylogenetic analysis revealed that isolates were distributed broadly, with the greatest proportion coming from phylogroup B2 (29.3% 24/82), followed by phylogroups D (25.6% 21/82), F (14.6% 12/82), A (12.1%, 10/82), C (9.8% 8/82), B1 (7.3% 6/82), and E (1.2% 1/82). Among the 82 CR *E. coli* isolates, 24 distinct STs were identified and 5 of these 24 were unknown STs. ST131 was the dominant ST, accounting for 19.5% (17/82), followed by ST405 (14.6%, 12/82) (see Table 1). Other pandemic STs, such as ST69 (4.9%, 4/82), ST10 (3.7%, 3/82), ST95 (1.2%, 1/82), and ST73 (1.2%, 1/82), were less prevalent.

**Figure 1:**
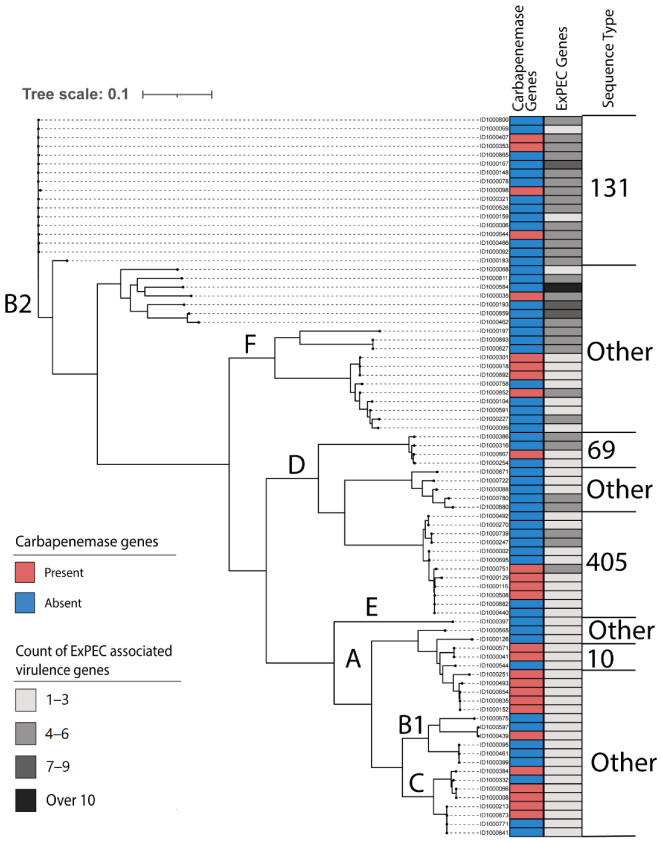
Maximum likelihood phylogenetic tree of 82 carbapenem resistant *E. coli* isolates from Alameda County Health systems 2017–2019, derived from a core alignment of 18,587 core SNP’s. The tree was generated using the general time reversible model with FigTree. A, B1, B2, C, D, E, and F are insilico ClermonTyper phylogenetic groups. The presence of any carbapenemase genes and the count of ExPEC virulence genes per isolate are indicated. The pandemic sequence types are annotated. “Other” sequence types include all sequence types other than ST131, ST69, ST405, and ST10 detected in the sample group.

Further subtyping was performed to identify the relationships between isolates of the same ST. Among ST131, 16 isolates belonged to the *fim*H30 subtype, and one to *fim*H41. All isolates from ST405 and ST69 were subtyped to *fim*H27. Whole genome SNPs were used to determine the diversity and evolutionary relationships of the carbapenem-resistant (CR) *E. coli* isolates (Figure 1). The analysis revealed diverse STs, but those identified were limited to specific phylogroups.

There was a moderate Pearson correlation between ST44 and the collection category of other (r = 0.50, see Figure S1). The other category represents specimen sources not related to urine, blood, skin, and stool/rectal swabs and other bodily fluids such as sputum, peritoneal fluid, and dialysate fluid. There was also a moderate correlation between ST90 and the collection category of other (r = 0.42) and ST73 and blood specimen sampling (r = 0.51).

### 2.2. Distribution of Antibiotic Resistance Genes

In total, 71 ARGs representing resistance to nine different classes of antibiotics were present across the CR-*E. coli* sequenced. The resistance profile for the isolate pool was diverse and comprised of five genes for carbapenemases from Ambler classes: One isolate contained *bla*_KPC,_ a Class A gene; 20 isolates contained class B *bla*_NDM_ family genes, and six contained *bla*_OXA_ family class D genes (see Table 2). Carbapenem resistance gene *bla*_NDM-5_ was highly prevalent within ST405, ST167, and ST6870; likewise, *bla*_OXA-181_ was most often found within ST410. ST131 contained the broadest diversity of carbapenemase genes. All four of the ST90 isolates originated from the same patient; however, only two of the ST90 isolates contained *blaNDM-1* carbapenemase-resistance genes (see Supplementary Table S3).

Seventy-four isolates (90.2%) contained genes for β-lactamases from either the CTX, CMY, SHV, CTX, or TEM (see Supplementary Table S1). CTX-M β-lactamase genes were present in over half of the isolates (53.6%). Within the CTX-M family, *bla*_CTX-M-15_ was predominant (35.4% of all isolates). All ST131 isolates contained genes from the CTX-M family, more than half belonging to *bla*_CTX-M-15_. Similarly, more than half the ST405 isolates contained *bla*_CTX-M-15_. *bla*_TEM-1B_ was found in 28 isolates (34.1%), and *bla*_OXA-1_ was present in 25 isolates (30.1%). A total of 19 isolates contained both *bla*_OXA-1_ and *bla*_CTX-M-15_. The results from the chi-squared analysis suggested an association between *bla*_OXA-1_ and *bla*_CTX-M-15_ (see Supplementary Table S1). The multi-drug transporter gene *mdf*(A)_1, which provides resistance to ethidium bromide, tetraphenylphosphonium, rhodamine, daunomycin, benzalkonium, rifampin, tetracycline, and puromycin as well as chloramphenicol, erythromycin, certain aminoglycosides, and fluoroquinolones, was present in all isolates (100%) [23]. Aminoglycoside resistance genes were highly prevalent, with one of the 12 genes being present in 65 isolates (79.3%) at least once. There were four other categories of dominant resistance genes. Tetracycline resistance genes for either *tet*(A)_6 or *tet*(B)_2 were present in 42 isolates (51.2%). Sulfonamide resistance genes *sul1_5* and *sul2_2* were present in 43 (52.4%) and 27 (33.0%) of the isolates respectively. A broad range of Trimethoprim resistance genes, *dfrA12_8*, *dfrA14_5*, and *dfrA17_1*, were individually present in at least one of the 48 isolates (58.5%). Furthermore, macrolide resistance gene *mph*(A)_2 was present in 32 isolates (39%), although six other macrolide resistance genes (*erm*(B)_1, *erm*(B)_18, *mef*(C)_1, *mph*(B)_1, *mph*(E)_1, *mph*(G)_1, and *msr*(E)_1) were present in 16 isolates. Although less pervasive, chloramphenicol resistance existed in 11 isolates primarily through the *flo*R_2 gene; while fluoroquinolone resistance predominantly from the *qnr*S1_1 gene, contained in eight isolates, was also present in a total of 11 isolates (see Table S1).

Resistance genes for third generation cephalosporins, *bla*_CTX-M-15_ and *bla*_TEM-1B_, were found in most STs (41.6% and 62.5%). Furthermore, aminoglycoside resistance was found in more than half the STs (58.3%) via the streptomycin resistance genes *aph*(6)-Id_1 and *aph*(3″)-Ib_5. When observing the total number of ARGs by ST, both STs 131 and 405 contained the broadest class range and largest number of ARGs. Among ST131s, the maximum number of ARGs present was 13, with a minimum of 2, an SD of 4.04, and an IQR of 7. Similarly, in ST405 isolates, there was a maximum of 17 ARGs, the highest observed number of ARGs in any isolate, with a minimum of 1, a standard deviation of 4, and an IQR of 7. STs 95, 73, 1193, 144, and 122 contained fewer ARGs than the median ARG count of eight. Of note, ST95 contained one ARG, while ST73 contained two ARGs.

### 2.3. Distribution of Virulence Genes

In total, 560 virulence genes were identified within the 82 *E. coli* analyzed. Adhesins *fim*G, *fim*F, and *fim*D were present in 100% of the isolates. Other adhesins, notably the curli fiber expression genes *csgA*, *csgC*, and *csgG*, were present in most isolates (98%). Invasins *ibeB, nadA*, and *nadB* were also present in most isolates (98%). Siderophore VFGs including *entA* and *fepA* were present in 95% of the isolates, with fuyA present in 78% of the isolates. Enterotoxigenic *E. coli* VFG *eae*H was present in 99% of the isolates, with the related *eaeX* being present in 13%.

**Figure 2:**
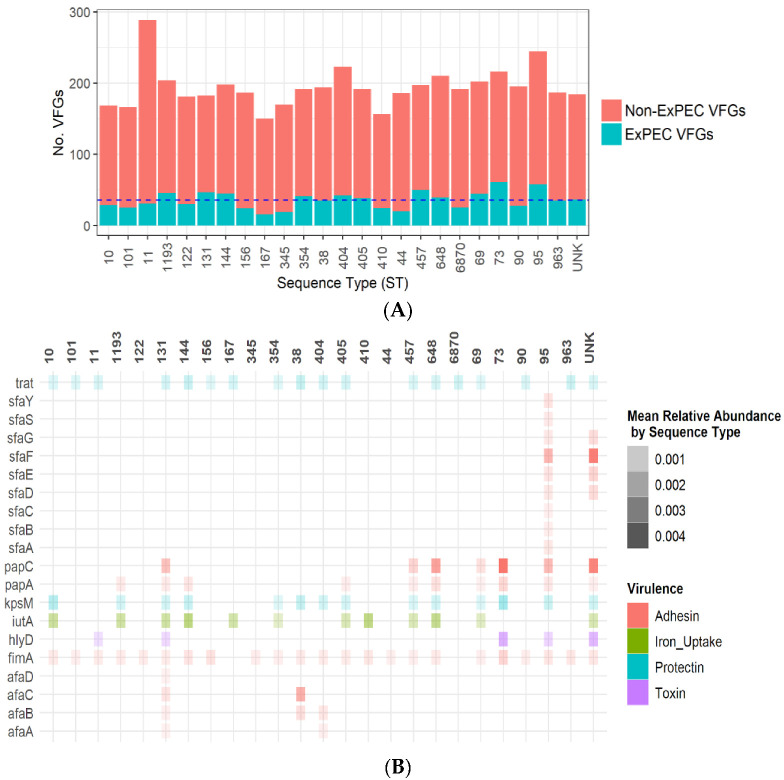
Distribution of putative extraintestinal pathogenic *E. coli* (ExPEC) virulence factor genes (VFGs) compared to non-ExPEC associated VFGs among *E. coli* STs from Alameda County, CA hospital patients from 2017–2019. (**A**) Putative ExPEC virulence genes representing 19 sub types from seven virulence gene families (*afa, fim, hlyD, iutA, kpsM, traT, pap*, *sfa*) were selected for sub-analysis. The average number of ExPEC VFGs among all STs was calculated and displayed as the dashed blue y-intercept line, with a value of 35.68 ExPEC VFGs per ST. (**B**) The relative abundance of each virulence gene is the total length of the virulence gene sequence divided by the total genome sequence length. The mean value of relative abundance was calculated by ST.

Putative ExPEC genes for adhesins (all of the *afa* family, *fim*A, *pap*A, and *pap*C, and all of the *sfa* family), siderophore (*iut*A), protectins (*kps*M and *traT*), and the toxin *hyl*D were selected for further analysis based on results obtained by Johnson et al. [8]. The total number of VFGs among isolates was a median of 190 VFGs per isolate, with a smaller proportion of genes attributed to ExPEC-related VFGs. As shown in Figure 2A, the absolute mean of ExPEC VFGs in pandemic lineages varied greatly: ST 131 (46.58 mean, SD 3.85); ST69 (44.25 mean, SD 7.69); ST 73 (61, single isolate); ST405 (38.25 mean, SD 3.37); and ST95 (58, single isolate). In Figure 2A, 24 sequence types (STs) were identified and one unknown ST. The distribution of putative ExPEC genes by ST is shown in Figure 2B, with the VFGs *fim*A (92%), *tra*t (71%), *kps*M (54%), and *iut*A (46%) being present in most STs.

In Figure 2B, putative ExPEC virulence genes were selected for analysis, with these representing 20 sub types from seven virulence gene families (*afa*, *fim*, *hlyD*, *iutA*, *kpsM*, *traT*, *pap*, *sfa*) within 24 STs and one unknown ST category. ST131 contained multiple *afa* adhesin genes, while ST95 contained numerous *sfa* adhesin genes. The gene *tra*T, which is commonly associated with ExPEC pathotypes, was present in 71% of the STs [24,25,26].

A Welch two-sample t-test determined that there was a statistically significant difference in the mean number of VFGs between pandemic and non-pandemic ST lineages (p = 0.035). There was no significant correlation between VFGs and ARGs occurring in the same isolates p > 0.05, with numerous weak negative correlation coefficients, the largest being r = −0.042 for papA related to dfrA12 and r = −0.042 for papA and aaDA2. There was no significant correlation between VFG and isolate sample source sources of urine, blood, skin, stool/rectal swabs, and other bodily fluids such as sputum, peritoneal fluid, and dialysate fluid, or other sources.

There were seven instances where more than one isolate was collected from the same patient. In all seven cases, the ST were identical among isolates originating from each patient, and the was no difference in the putative ExPEC gene profile when isolates from the same patient were compared. However, the total virulence gene profile varied from organism to organism. Although horizontal gene transfer is difficult to detect, we saw evidence of clonal variation in patients with multiple isolate samples [21,22].

## 3. Discussion

Our results provide a comprehensive genotypic characterization of carbapenem resistant *E. coli* that were present in carbapenem-resistant infections in Alameda County, California between 2017 and 2019. Drug-resistant infections in Alameda were caused by a broad set of *E. coli* phylogroups. This is different than what has been reported previously with extended spectrum β-lactamase (ESBL) infections, which suggests that only the phylogroups B2 and D are of primary importance [10,19,27]. Furthermore, a molecular analysis of our data revealed the presence of several pandemic strains (e.g., ST131, ST69, ST95, and ST73), which have been identified across the globe, as well as ST405 and ST10, which have been identified as rapidly increasing in prevalence and distribution [11,12]. However, our analysis also demonstrated a significant amount of variation in STs, which was unexpected. Although a recent review of ExPECs identified a similarly broad range of global STs contributing to the global burden of disease [28], other studies evaluating global and regional carbapenem-resistant *E. coli* have limited the STs to a small number [29,30]. Our study demonstrates the rapid expansion of carbapenem resistance among a broader range of STs within Alameda County, California.

The breadth of *E. coli* STs as producers of all Ambler class carbapenemases is noteworthy and potentially reflects the important role of *E. coli* in the dissemination of carbapenemases within the region. The literature has identified ST131 as the major cause of serious multidrug-resistant *E. coli* infections globally [28,31]. Our study found that within ST131, there was large variation in the carbapenemase genes present, including classes A, B and D; class C was not present. The spectrum of carbapenemase genes present in the ST 131 isolates shows their ability to incorporate important ARGs and is likely associated with their capacity to cause disease [4,32]. Among the carbapenemase genes present, the distribution of these clinically important enzymes was limited to 33% of the ST 131 isolates. These results are slightly lower than the results from a similar Northern California study analyzing carbapenemase resistance between 2013 and 2016 but may be attributed to a much smaller sample size (*n* = 24) [33]. The discrepancy in phenotypic and genotypic carbapenem resistance is potentially explained by alternative genetic mechanisms such as chromosomal mutations, drug efflux pumps, porins [34,35], or the overexpression of the extended spectrum β-lactamase and ampC β-lactamase genes [36]. This discrepancy is highlighted by a group of four isolates, all of which are ST90, which were collected from the same patient and included in this study. Two isolates contained *blaNDM-5* resistance genes, while two isolates contained no resistance genes for carbapenemase resistance (see Supplementary Table S3). Further, in-depth analyses of resistance determinants would clarify the full extent of each of these mechanisms in mediating carbapenem resistance.

In total, 71 ARGs that encoded resistance to antibiotics, representing nine different classes, were identified. Seventy-four out of the 82 isolates contained genes for β-lactamases from the families of CTX, CMY, SHV, and TEM. The presence of both carbapenem resistance and third generation cephalosporin resistance in many isolates is indicative of both the historical impact of cephalosporin use [3] and the significance of this threat in the context of the increase in untreatable clinical infections [5,37]. Furthermore, studies have demonstrated that the multi drug transporter gene *mdf*(A) confers broad spectrum antibiotic resistance [18,22]. Our study found that the *mdf*(A) gene was present in every isolate, and each isolate also contained numerous other resistance genes. Therefore, the resistance profile of each isolate was extremely broad. The presence of such a broad range of resistance genes among a diverse pool of STs underscores the adaptability of *E. coli* pathotypes and constitutes a significant threat to human health due to the limitations medical providers would face when searching for treatment options for these infections [28,38].

The most common ESBL genes globally are the *bla*_CTX-M_ genes, of which CTX-M-15 is one of the predominant allelic variants. The literature indicates a significant increase in the global distribution of and subsequent rise in ESBL infections [32,39]. Our study found that over half of the *E. coli* contained CTX-M genes. Furthermore, all ST131 isolates contained *bla*_CTX-M_, with over half being *bla*_CTX-M-15_, which is in line with recent studies that have demonstrated ST131 are strongly associated with *bla*_CTX-M-15_ [28,31,40,41]. The relationship between ST131 and *bla*_CTX-M-15_ is noteworthy due to the high burden of disease attributable to ST131 [5,31]. As the global prevalence of ESBLs and ESBL-producing *Enterobacterales* infections continue to rise, the spread of ESBL genes will likely drive an increase in prescriptions for carbapenems and therefore an increase in carbapenemase genes, threatening the long-term effectiveness of this last-line antibiotic.

This study also identified 19 isolates that contained both *bla*_OXA-1_ and *bla*_CTX-M-15_, with a significant correlation among four STs: ST131, ST405, ST345, and ST167. The co-carriage of *bla*_OXA-1_ and *bla*_CTX-M-15_ has been previously identified in ST131 [42] and ST405 [43]; however, our study also showed co-carriage in ST345 and ST167, which had not been previously identified. Previous studies have demonstrated that the co-carriage of *bla*_OXA-1_ and *bla*_CTX-M-15_ is mediated through co-location on a plasmid [42,43]. This study did not evaluate plasmid composition, and this represents a limitation of the analysis.

A key feature of ExPEC relates to the high virulence capacity of this organism [44,45]. The efficiency of mobile genetic elements when it comes to transferring VFGs facilitates the dissemination and collection of virulence factors among ExPEC strains [30]. Among the 82 *E. coli* isolates in our analysis, there were 560 unique VFGs. VFGs allow the organism to survive, adapt, and evade the host environment and defense mechanisms [20]. The number of VFGs within pandemic ST lineages was significantly higher than non-pandemic lineages and is a possible correlation to the increased pathogenicity of the pandemic STs. Putative ExPEC genes such as *fim*A, *kps*M, and *iut*A were present in most STs, indicating the highly virulent isolates and the breadth of plasticity within these bacteria. These findings compliment recent studies which demonstrated that among isolates identified as ExPEC by putative ExPEC genes, there were many VFGs present which aid the bacteria in causing a broad range of infections [10].

Our study aligns with the work of Johnson et al. and Hung [46,47], who determined that iron uptake gene *fyuA* has a higher prevalence than *iutA* and that protectin *traT* has a higher prevalence than *kpsM*. The current virulence factor gene bank used as definitive markers for ExPECs is based upon a large dataset originating primarily from phylogroups B2 and D [48,49,50]. Although these two groups are hypothesized as the source of horizontal transfer among *E. coli*, our study demonstrates that the current breadth of clinically relevant carbapenem-resistant ExPECs has expanded far beyond these two “original” phylogroups. This raises concern for establishing pathogenesis based upon virulence parameters which may only represent a distinct subset of the pathotype. Of the ExPEC pathotypes, certain virulence genes are attributed to specific pathotypes and represent the adaptation to colonize a specific niche. VFGs such as the iron uptake genes *fyuA*, *chu*A, and the proteolytic toxin gene *vat* are associated with UPEC [47,51], while the porin *omp*A gene and invasin *ibe*A are associated with NMEC [45,52]. Several of our isolates contained VFGs that are related to separate ExPEC pathotypes, which conflicts with the current literature that bases pathotype identification only on virulence factor analysis. Other studies also identified similar trends in heterogeneity of NMEC virulence factors which conflict with pathotyping techniques [53,54]. Furthermore, each isolate from our study that deviated from common pathotype virulence comparisons were from unique STs. The overlap of ExPEC pathotype-related VFGs underscores the challenges of using a putative gene method to identify ExPEC pathotypes. One possible confounder of this typing method could be the overlap between commensal intestinal colonization and extraintestinal virulence. Recent studies have identified numerous virulence factors which are present in both commensal and pathogenic *E. coli* [29,55]. Further investigation of associated mechanisms and the inclusion of other markers such as resistance phenotypes and clinical syndromes is required to increase the predictive power of virulence factors for pathotypes.

Although ExPEC pathotyping by virulence factors lacks definition, there is a strong body of evidence for the use of VFGs in pathotyping enteropathogenic *E. coli* (EPEC) and enterotoxin *E. coli* (ETEC) [56,57]. Highly conserved *eae*H and *eae*X genes, which are used as key identifying markers for the pathotypes, were present in almost all our study isolates. This finding may represent a relationship between EPEC and ExPEC, explained by their transient colonization of the gut and then exposure to sterile sites, as opposed to their commensal origin, which does not carry the VFGs identified [58].

This study did not include all *Enterobacterales* species, and an evaluation of ARGs, VFGs, and plasmids among different *Enterobacterales* species would greatly improve our understanding of the spread of antibitoic resistance and the mechanisms involved. Additional analyses of the plasmids would be helpful in understanding the genetic elements driving the spread. The identification of ARGs and VFG locations—either chromosomally, via plasmids or both—is essential for understanding the horizontal gene transfer mechanisms of these organisms. Another limitation is the lack of clinical data that could help us to link isolate epidemiologically and help to pinpoint the potential origin of infection and spatial relationships. Although a data-use agreement is in the process of being completed, this study would have benefited greatly from background information pertaining to the clinical isolates other than the date range, isolate derivation from the inpatient hospital, skilled nursing facilities, or long-term acute care facilities, and source type.

The purpose of this study was to enhance the understanding of clinically important drug-resistant *E. coli* that are present in Alameda County, California, and it has described the most common types of virulence and resistance genes. Moreover, this study has demonstrated a high prevalence of CTX-M-mediated resistance. The presence of CTX-M genes will inevitably lead to increased carbapenem use in humans, with an associated rise in resistance. Understanding temporal trends in these genotypes will help in the development of hypotheses as to why these changes occur, supporting strategies for reducing the spread of AMR in Gram-negative bacteria, averting excess mortality, and preserving existing classes of antibiotics for future generations.

## 4. Conclusions

Carbapenem resistance is a rising concern for public health departments. Our study has characterized the genomic features of *E. coli* isolates responsible for carbapenem resistant infections in Alameda County from 2017 to 2019. In contrast to previous findings, our data suggest that a broad range of phylogroups and sequence types are responsible for these infections, rather than only the traditional high risk clonal groups (e.g., ST131, ST69, and ST10). A broad repertoire of carbapenemase genes from each Ambler class were in circulation in the Alameda County health facilities, in addition to ESBL genes including *bla*_CTX-M-15_. Over 500 known virulence genes were detected within the 82 isolates characterized, and at least one ExPEC-associated virulence gene was detected in all isolates. There was a higher prevalence in terms of virulence genes in pandemic ST lineages. The heterogeneity of clonal group-associated resistance genes and virulence genes detected in our analysis suggests the high adaptability and diversity of *E. coli* pathotypes in the community. This will present an ongoing challenge for public health mitigation and will demand broad genomic surveillance.

## 5. Materials and Methods

A total of 82 carbapenem-resistant *E. coli* isolates were received by the Alameda County Public Health Laboratory (ACPHL) between June 2017 and July 2019. All isolates were obtained from Clinical Laboratory Improvement Amendments (CLIA) certified clinical laboratories within the county of Alameda and originated from individual patients. Seven patients had multiple isolates due to special circumstances, such as facility transfer or variation in the isolate susceptibility profiles (see Supplementary Table S2). Isolates were derived from inpatient hospitals, skilled nursing facilities, or acute care facilities.

### 5.1. Susceptibility Testing

All of the healthcare facility labs that supplied isolates to ACPHL were CLIA certified. Most labs performed susceptibility testing using the minimum inhibitory concentration (MIC) method; however, a small number of facilities use the Kirby–Bauer disk diffusion method. All isolates were determined to be carbapenem-resistant according to the CLSI M100 guidelines, and CLSI breakpoints for ertapenem, imipenem, and meropenem were used. *E. coli* ATCC 25922 was used as a control strain.

### 5.2. Whole Genome Sequencing

Purified DNA was extracted from each *E. coli* isolate for whole-genome sequencing and subsequent genomic analyses. DNA extraction was performed by ACPHL using a Roche MagNa Pure Compat Instrument (Roche, Basel, Switzerland), in accordance with the manufacturer’s protocols. Purified DNA was quantified by fluorimetry using Qubit 3.0 (Invitrogen, Carlsbad, CA, USA).

ACPHL performed library preparation for whole-genome sequencing by starting with the quantified DNA diluted to 0.2 ng/µL which was then fragmented and tagged using a Nextera XT Library Preparation Kit (Illumina, San Diego, CA, USA). Indexed Libraries were purified and quantified for quality using a Qubit 3.0 fluorometer in combination with their Qubit negative and Qubit positive broad range standards. Samples were pooled in equimolar quantities and subjected to DNA sequencing using single-end, 150-cycle reactions in a MiSeq (Illumina, San Diego, CA, USA) at ACPHL.

The Illumina dual-indexed, single-read sequences were assembled using Unicycler, a SPAdes assembly pipeline with polishing steps using Pilon, Bowtie2, and Samtools [59]. De-novo assembly was performed for all bacterial isolate genomes using sequence reads with a minimum length of 500 bp.

Each sequencing run procedure, starting from DNA extraction through sequencing and sequencing analysis, included the isolate BAA-2146 (ATCC) as a control. BAA-2146 is a clone of *Klebsiella pneumoniae* which has been completely sequenced and its plasmid content fully characterized. Library prep and the sequencing process included a final FASTA file of BAA-2146, which was annotated for specific genes distributed throughout the chromosome and plasmids as a means of quality control.

### 5.3. Genotypic Analysis

The ACPHL performed species identification on GAMBIT software. The GAMBIT reference database contains over 50,000 genome sequences representing 1445 bacterial species compiled and curated from the National Center for Biotechnology Information’s Reference Sequence Collection, which includes over 4000 genome sequences from *E. coli*.

Phylogroup analysis of the *E. coli* genomes was performed using in silico ClermonTyper [60,61]. Multi-locus sequence typing was performed on all sequences using seven housekeeping genes, *adk*, *fumC*, *gyrB*, *icd*, *mdh*, *purA*, and *recA*, by cross referencing the isolate sequence types with the Center for Genomic Epidemiology MLST 2.0 database that makes use of MLST allele sequences and profile data from PubMLST.org [62]. *E. coli* sub typing using *fim*H single nucleotide polymorphism (SNP) analysis was also performed on all *E. coli* sequences using the FimTyper-1.0 database from the Center for Genomic Epidemiology [63] with a threshold ID of 95%.

The sequence data for the 82 *E. coli* genomes were screened to identify antibiotic resistance genes (ARGs) and VFGs using the software ABRicate [64], a mass screening tool for identifying contigs. ABRicate software integrates the ResFinder 4.0 database [65] and Virulence Finder database (VFDB) [66]. ResFinder search parameters were set to the default: 90% sequence identity with a minimum sequence overlap length of 60%. VFDB BLAST search parameters were set to default using the nucleotide sequences from the VFDB core dataset (setA) database and the blastn program: low complexity filter, Expect 0.01, and Matrix BLOSUM62.

For phylogenetic analyses, Snippy v3.2 [67] was used to map reads to the *E. coli* MG1655 reference genome (NCBI:txid511145). The SNP distance between isolates was calculated using snp-dists v0.8.2 [68]. FastTree v2.0 was used to generate an approximate maximum likelihood phylogenetic tree using the general time reversible model. The tree was visualized in iTol v6 [69].

### 5.4. Statistical Analysis

Chi square tests for independence, with a significance level of *alpha* = 0.05, were used to assess the independence between genes. A Welch two sample *t*-test was used to evaluate the difference between the means of virulence factor genes between pandemic and non-pandemic lineages using a significance level of *alpha* = 0.05. To evaluate if there was any correlation between the presence of ARGs and VFGs, a Pearson correlation coefficient was calculated to evaluate the correlation between the presence of VFGs and ARGs among isolates. A Pearson correlation coefficient was calculated to evaluate the correlation between ST and Isolate specimen source as well as VFG and Isolate specimen source. To show the amount of genetic material within the genomes made up by specific genes in relation to the whole genome, the relative abundance was calculated. The relative abundance was calculated by taking the total length of the ARG sequence and dividing it by the total genome length for each isolate. The ST relative abundance was calculated by taking the mean of the relative abundance for all the isolates within the ST group. All statistical analyses were performed using R Studio software version 1.4.1103 with the following R packages: ggplot for visualization [70] and dplyr [71], stringr [72], tidyr [73], knitr [74], kableExtra [75], and fastDummies [76] for data manipulation and reporting.

## Figures and Tables

**Table 1:** Distribution and prevalence MLST and fimH types in groups of *CRE* isolates.

Phylogroup	*fim*H Type	Sequence Type	Prevalence (*n =* 82)
A	*fim*H27	ST10	1
	*fim*H54	ST10	2
		ST44	1
		ST UNK1 ^a^	1
	*fim*H Absent	ST167	5
B1	*fim*H31	ST345	3
	*fim*H38	ST156	2
	*fim*H191	ST101	1
B2	*fim*H10	ST73	1
	*fim*H18	ST95	1
	*fim*H21	ST122	1
	*fim*H27	ST404	1
	*fim*H30	ST131	16
	*fim*H41	ST131	1
	*fim*H54	ST144	1
	*fim*H64	ST1193	1
	*fim*H120	ST UNK2 ^b^	1
C	*fim*H24	ST410	4
	*fim*H142	ST90	4
D	*fim*H5	ST38	1
	*fim*H26	ST963	1
	*fim*H27	ST69	4
		ST405	12
	*fim*H54	ST38	1
	*fim*H65	ST38	1
		ST UNK3 ^c^	1
E	*fim*H82	ST11	1
F	*fim*H5	ST648	1
	*fim*H27	ST648	2
	*fim*H171	ST648	1
	*fim*H145	ST457	1
	*fim*H58	ST354	2
	*fim*H Absent	ST6870	3
	*fim*H Absent	ST UNK4 ^d^/5 ^e^	2

^a,b,c,d,e^ Identifies five distinct unknown sequence types (STs).

**Table 2:** Distribution and prevalence of the most common Ambler Class Carbapenemases by MLST from Alameda County, CA hospital patients from 2017–2019.

Ambler Class	Carbapenemase Gene	Sequence Type	Isolates (*n* = 82)
A	*bla*KPC-2	ST131	1
B	*bla*NDM-1	ST131	2
		ST90	2
	*bla*NDM-5	ST167	5
		ST405	4
		ST6870	3
		ST10	2
		ST69	1
		ST73	1
D	*bla*OXA-181	ST410	3
		ST156	1
		STUNK5 *	1
	*bla*OXA-48	ST131	1

* STUNK5 represents unidentified ST based on MLST methods.

## Data Availability

The data presented in this study are openly available in at NCBI Genbank, bioproject ID: PRJNA870932, accession numbers SAMN30393057 through SAMN30393138, https://www.ncbi.nlm.nih.gov/Traces/wgs/?page=1&view=wgs&search=PRJNA870932.

## Supplementary Materials

The following supporting information can be downloaded at: https://www.mdpi.com/article/10.3390/antibiotics11121794/s1, Figure S1: Pearson Correlation Coeficcients of Isolate collection meta-data from Alameda County, California, 2017–2019; Table S1: Summary of antibiotic resistance gene frequencies in CRE isolates, Table S2: Summary of Isolate collection meta-data from Alameda County, California, 2017–2019, Table S3: Isolate meta data, ST type and Carbapenemase Resistance Genes from Isolates collected from the same patients from Alameda County, California, 2017–2019.

## References

[1] Logan L.K., Weinstein R.A. (2017). The Epidemiology of Carbapenem-Resistant Enterobacteriaceae: The Impact and Evolution of a Global Menace. J. Infect. Dis..

[2] Guh A.Y., Limbago B.M., Kallen A.J. (2014). Epidemiology and Prevention of Carbapenem-Resistant Enterobacteriaceae in the United States. Expert Rev. Anti Infect. Ther..

[3] Rahal J.J., Urban C., Horn D., Freeman K., Segal-Maurer S., Maurer J., Mariano N., Marks S., Burns J.M., Dominick D. (1998). Class Restriction of Cephalosporin Use to Control Total Cephalosporin Resistance in Nosocomial *Klebsiella*. JAMA.

[4] Meletis G. (2016). Carbapenem Resistance: Overview of the Problem and Future Perspectives. Ther. Adv. Infect. Dis..

[5] Tacconelli E., Carrara E., Savoldi A., Harbarth S., Mendelson M., Monnet D.L., Pulcini C., Kahlmeter G., Kluytmans J., Carmeli Y. (2018). Discovery, research, and development of new antibiotics: The WHO priority list of antibiotic-resistant bacteria and tuberculosis. Lancet Infect. Dis..

[6] MacKinnon M.C., Sargeant J.M., Pearl D.L., Reid-Smith R.J., Carson C.A., Parmley E.J., McEwen S.A. (2020). Evaluation of the Health and Healthcare System Burden Due to Antimicrobial-Resistant *Escherichia coli* Infections in Humans: A Systematic Review and Meta-Analysis. Antimicrob. Resist. Infect. Control.

[7] Johnson T.J., Nolan L.K. (2009). Pathogenomics of the Virulence Plasmids of *Escherichia coli*. Microbiol. Mol. Biol. Rev..

[8] Johnson J.R., Johnston B., Clabots C., Kuskowski M.A., Castanheira M. (2010). *Escherichia coli* Sequence Type ST131 as the Major Cause of Serious Multidrug-Resistant *E. Coli* Infections in the United States. Clin. Infect. Dis..

[9] Biran D., Ron E.Z., Frankel G., Ron E.Z. (2018). Extraintestinal Pathogenic *Escherichia coli*. Escherichia coli, a Versatile Pathogen.

[10] Sarowska J., Futoma-Koloch B., Jama-Kmiecik A., Frej-Madrzak M., Ksiazczyk M., Bugla-Ploskonska G., Choroszy-Krol I. (2019). Virulence Factors, Prevalence and Potential Transmission of Extraintestinal Pathogenic *Escherichia coli* Isolated from Different Sources: Recent Reports. Gut Pathog..

[11] Riley L.W. (2014). Pandemic Lineages of Extraintestinal Pathogenic *Escherichia coli*. Clin. Microbiol. Infect. Off. Publ. Eur. Soc. Clin. Microbiol. Infect. Dis..

[12] Manges A.R., Geum H.M., Guo A., Edens T.J., Fibke C.D., Pitout J.D.D. (2019). Global Extraintestinal Pathogenic *Escherichia coli* (ExPEC) Lineages. Clin. Microbiol. Rev..

[13] Pitout J.D.D., DeVinney R. (2017). *Escherichia coli* ST131: A Multidrug-Resistant Clone Primed for Global Domination. F1000Research.

[14] Pitout J.D., Laupland K.B. (2008). Extended-Spectrum β-Lactamase-Producing Enterobacteriaceae: An Emerging Public-Health Concern. Lancet Infect. Dis..

[15] Alhashash F., Weston V., Diggle M., McNally A. (2013). Multidrug-Resistant *Escherichia coli* Bacteremia. Emerg. Infect. Dis..

[16] El Salabi A., Walsh T.R., Chouchani C. (2013). Extended Spectrum β-Lactamases, Carbapenemases and Mobile Genetic Elements Responsible for Antibiotics Resistance in Gram-Negative Bacteria. Crit. Rev. Microbiol..

[17] Suay-García B., Pérez-Gracia M.T. (2019). Present and Future of Carbapenem-Resistant Enterobacteriaceae (CRE) Infections. Antibiotics.

[18] Codjoe F.S., Donkor E.S. (2017). Carbapenem Resistance: A Review. Med. Sci..

[19] Johnson J.R., Russo T.A. (2002). Extraintestinal Pathogenic *Escherichia coli*: “The Other Bad *E. Coli*”. J. Lab. Clin. Med..

[20] Kaper J.B., Nataro J.P., Mobley H.L.T. (2004). Pathogenic *Escherichia coli*. Nat. Rev. Microbiol..

[21] Arnold B.J., Huang I.-T., Hanage W.P. (2022). Horizontal Gene Transfer and Adaptive Evolution in Bacteria. Nat. Rev. Microbiol..

[22] Brito I.L. (2021). Examining Horizontal Gene Transfer in Microbial Communities. Nat. Rev. Microbiol..

[23] Edgar R., Bibi E. (1997). MdfA, an *Escherichia coli* Multidrug Resistance Protein with an Extraordinarily Broad Spectrum of Drug Recognition. J. Bacteriol..

[24] Rezatofighi S.E., Mirzarazi M., Salehi M. (2021). Virulence Genes and Phylogenetic Groups of Uropathogenic *Escherichia coli* Isolates from Patients with Urinary Tract Infection and Uninfected Control Subjects: A Case-Control Study. BMC Infect. Dis..

[25] Tanabe R.H.S., Dias R.C.B., Orsi H., de Lira D.R.P., Vieira M.A., dos Santos L.F., Ferreira A.M., Rall V.L.M., Mondelli A.L., Gomes T.A.T. (2022). Characterization of Uropathogenic Escherichia Coli Reveals Hybrid Isolates of Uropathogenic and Diarrheagenic (UPEC/DEC) *E. coli*. Microorganisms.

[26] Gultekin E.O., Ulger S.T., Delialioğlu N. (2022). Distribution of Pathogenicity Island Markers and Virulence Factors Genes of Extraintestinal Pathogenic *Escherichia coli* Isolates. Jundishapur J. Microbiol..

[27] Koga V.L., Tomazetto G., Cyoia P.S., Neves M.S., Vidotto M.C., Nakazato G., Kobayashi R.K.T. (2014). Molecular Screening of Virulence Genes in Extraintestinal Pathogenic *Escherichia coli* Isolated from Human Blood Culture in Brazil. BioMed Res. Int..

[28] Pitout J.D. (2012). Extraintestinal Pathogenic *Escherichia coli*: An Update on Antimicrobial Resistance, Laboratory Diagnosis and Treatment. Expert Rev. Anti Infect. Ther..

[29] Diard M., Garry L., Selva M., Mosser T., Denamur E., Matic I. (2010). Pathogenicity-Associated Islands in Extraintestinal Pathogenic *Escherichia coli* Are Fitness Elements Involved in Intestinal Colonization. J. Bacteriol..

[30] Dobrindt U., Chowdary M.G., Krumbholz G., Hacker J. (2010). Genome Dynamics and Its Impact on Evolution of *Escherichia coli*. Med. Microbiol. Immunol..

[31] Johnson J.R., Delavari P., Kuskowski M., Stell A.L. (2001). Phylogenetic Distribution of Extraintestinal Virulence-Associated Traits in *Escherichia coli*. J. Infect. Dis..

[32] Ludden C., Decano A.G., Jamrozy D., Pickard D., Morris D., Parkhill J., Peacock S.J., Cormican M., Downing T. (2020). Genomic Surveillance of *Escherichia coli* ST131 Identifies Local Expansion and Serial Replacement of Subclones. Microb. Genom..

[33] Senchyna F., Gaur R.L., Sandlund J., Truong C., Tremintin G., Kültz D., Gomez C.A., Tamburini F.B., Andermann T., Bhatt A. (2019). Diversity of Resistance Mechanisms in Carbapenem-Resistant Enterobacteriaceae at a Health Care System in Northern California, from 2013 to 2016. Diagn. Microbiol. Infect. Dis..

[34] Black C.A., So W., Dallas S.S., Gawrys G., Benavides R., Aguilar S., Chen C.-J., Shurko J.F., Lee G.C. (2021). Predominance of Non-Carbapenemase Producing Carbapenem-Resistant *Enterobacterales* in South Texas. Front. Microbiol..

[35] Zou H., Xiong S.-J., Lin Q.-X., Wu M.-L., Niu S.-Q., Huang S.-F. (2019). CP-CRE/Non-CP-CRE Stratification and CRE Resistance Mechanism Determination Help in Better Managing CRE Bacteremia Using Ceftazidime–Avibactam And Aztreonam–Avibactam. Infect. Drug Resist..

[36] Patidar N., Vyas N., Sharma S., Sharma B. (2021). Phenotypic Detection of Carbapenemase Production in Carbapenem-Resistant Enterobacteriaceae by Modified Hodge Test and Modified Strip Carba NP Test. J. Lab. Physicians.

[37] Seyedjavadi S.S., Goudarzi M., Sabzehali F. (2016). Relation between BlaTEM, BlaSHV and BlaCTX-M Genes and Acute Urinary Tract Infections. J. Acute Dis..

[38] Braz V.S., Melchior K., Moreira C.G. (2020). *Escherichia coli* as a Multifaceted Pathogenic and Versatile Bacterium. Front. Cell. Infect. Microbiol..

[39] Bevan E.R., Jones A.M., Hawkey P.M. (2017). Global Epidemiology of CTX-M β-Lactamases: Temporal and Geographical Shifts in Genotype. J. Antimicrob. Chemother..

[40] Petty N.K., Zakour N.L.B., Stanton-Cook M., Skippington E., Totsika M., Forde B.M., Phan M.-D., Moriel D.G., Peters K.M., Davies M. (2014). Global Dissemination of a Multidrug Resistant *Escherichia coli* Clone. Proc. Natl. Acad. Sci. USA.

[41] Yang Z., Sun Q., Chen S., Ding S., Zhang R., Zhu K. (2020). Genomic and Phenotypic Analysis of Persistent Carbapenem-Resistant *Klebsiella pneumoniae* Isolates from a 5-Year Hospitalized Patient. Microb. Drug Resist..

[42] Livermore D.M., Day M., Cleary P., Hopkins K.L., Toleman M.A., Wareham D.W., Wiuff C., Doumith M., Woodford N. (2019). OXA-1 β-Lactamase and Non-Susceptibility to Penicillin/β-Lactamase Inhibitor Combinations among ESBL-Producing *Escherichia coli*. J. Antimicrob. Chemother..

[43] Alousi S., Salloum T., Arabaghian H., Matar G.M., Araj G.F., Tokajian S.T. (2018). Genomic Characterization of MDR *Escherichia coli* Harboring BlaOXA-48 on the IncL/M-Type Plasmid Isolated from Blood Stream Infection. BioMed Res. Int..

[44] Bielaszewska M., Dobrindt U., Gärtner J., Gallitz I., Hacker J., Karch H., Müller D., Schubert S., Alexander Schmidt M., Sorsa L.J. (2007). Aspects of Genome Plasticity in Pathogenic *Escherichia coli*. Int. J. Med. Microbiol..

[45] Khan N.A., Shin S., Chung J.W., Kim K.J., Elliott S., Wang Y., Kim K.S. (2003). Outer Membrane Protein A and Cytotoxic Necrotizing Factor-1 Use Diverse Signaling Mechanisms for *Escherichia coli* K1 Invasion of Human Brain Microvascular Endothelial Cells. Microb. Pathog..

[46] Johnson J.R., Johnston B.D., Porter S., Thuras P., Aziz M., Price L.B. (2018). Accessory Traits and Phylogenetic Background Predict *Escherichia coli* Extraintestinal Virulence Better Than Does Ecological Source. J. Infect. Dis..

[47] Hung W.-T., Cheng M.-F., Tseng F.-C., Chen Y.-S., Shin-Jung Lee S., Chang T.-H., Lin H.-H., Hung C.-H., Wang J.-L. (2019). Bloodstream Infection with Extended-Spectrum Beta-Lactamase-Producing *Escherichia coli*: The Role of Virulence Genes. J. Microbiol. Immunol. Infect. Wei Mian Yu Gan Ran Za Zhi.

[48] Bingen E., Picard B., Brahimi N., Mathy S., Desjardins P., Elion J., Denamur E. (1998). Phylogenetic Analysis of *Escherichia coli* Strains Causing Neonatal Meningitis Suggests Horizontal Gene Transfer from a Predominant Pool of Highly Virulent B2 Group Strains. J. Infect. Dis..

[49] Boyd E.F., Hartl D.L. (1998). Chromosomal Regions Specific to Pathogenic Isolates of *Escherichia coli* Have a Phylogenetically Clustered Distribution. J. Bacteriol..

[50] Lecointre G., Rachdi L., Darlu P., Denamur E. (1998). *Escherichia coli* Molecular Phylogeny Using the Incongruence Length Difference Test. Mol. Biol. Evol..

[51] Spurbeck R.R., Dinh P.C., Walk S.T., Stapleton A.E., Hooton T.M., Nolan L.K., Kim K.S., Johnson J.R., Mobley H.L.T. (2012). *Escherichia coli* Isolates That Carry Vat, FyuA, ChuA, and YfcV Efficiently Colonize the Urinary Tract. Infect. Immun..

[52] Huang S.H., Wan Z.S., Chen Y.H., Jong A.Y., Kim K.S. (2001). Further Characterization of *Escherichia coli* Brain Microvascular Endothelial Cell Invasion Gene IbeA by Deletion, Complementation, and Protein Expression. J. Infect. Dis..

[53] Logue C.M., Doetkott C., Mangiamele P., Wannemuehler Y.M., Johnson T.J., Tivendale K.A., Li G., Sherwood J.S., Nolan L.K. (2012). Genotypic and Phenotypic Traits That Distinguish Neonatal Meningitis-Associated *Escherichia coli* from Fecal *E. coli* Isolates of Healthy Human Hosts. Appl. Environ. Microbiol..

[54] Wijetunge D.S.S., Gongati S., DebRoy C., Kim K.S., Couraud P.O., Romero I.A., Weksler B., Kariyawasam S. (2015). Characterizing the Pathotype of Neonatal Meningitis Causing *Escherichia coli* (NMEC). BMC Microbiol..

[55] Le Gall T., Clermont O., Gouriou S., Picard B., Nassif X., Denamur E., Tenaillon O. (2007). Extraintestinal Virulence Is a Coincidental By-Product of Commensalism in B2 Phylogenetic Group *Escherichia coli* Strains. Mol. Biol. Evol..

[56] Jerse A.E., Kaper J.B. (1991). The Eae Gene of Enteropathogenic Escherichia Coli Encodes a 94-Kilodalton Membrane Protein, the Expression of Which Is Influenced by the EAF Plasmid. Infect. Immun..

[57] Sheikh A., Luo Q., Roy K., Shabaan S., Kumar P., Qadri F., Fleckenstein J.M. (2014). Contribution of the Highly Conserved EaeH Surface Protein to Enterotoxigenic *Escherichia coli* Pathogenesis. Infect. Immun..

[58] Riley L.W. (2020). Distinguishing Pathovars from Nonpathovars: *Escherichia coli*. Microbiol. Spectr..

[59] Wick R.R., Judd L.M., Gorrie C.L., Holt K.E. (2017). Unicycler: Resolving Bacterial Genome Assemblies from Short and Long Sequencing Reads. PLOS Comput. Biol..

[60] Beghain J., Bridier-Nahmias A., Le Nagard H., Denamur E., Clermont O. (2018). ClermonTyping: An Easy-to-Use and Accurate in Silico Method for Escherichia Genus Strain Phylotyping. Microb. Genom..

[61] Clermont O., Dixit O.V.A., Vangchhia B., Condamine B., Dion S., Bridier-Nahmias A., Denamur E., Gordon D. (2019). Characterization and Rapid Identification of Phylogroup G in *Escherichia coli*, a Lineage with High Virulence and Antibiotic Resistance Potential. Environ. Microbiol..

[62] Jolley K.A., Maiden M.C. (2010). BIGSdb: Scalable Analysis of Bacterial Genome Variation at the Population Level. BMC Bioinform..

[63] Roer L., Tchesnokova V., Allesøe R., Muradova M., Chattopadhyay S., Ahrenfeldt J., Thomsen M.C.F., Lund O., Hansen F., Hammerum A.M. (2017). Development of a Web Tool for Escherichia Coli Subtyping Based on FimH Alleles. J. Clin. Microbiol..

[64] Seemann T. Tseemann/Abricate 2021. https://github.com/tseemann/abricate.

[65] Bortolaia V., Kaas R.S., Ruppe E., Roberts M.C., Schwarz S., Cattoir V., Philippon A., Allesoe R.L., Rebelo A.R., Florensa A.F. (2020). ResFinder 4.0 for Predictions of Phenotypes from Genotypes. J. Antimicrob. Chemother..

[66] Chen L., Yang J., Yu J., Yao Z., Sun L., Shen Y., Jin Q. (2005). VFDB: A Reference Database for Bacterial Virulence Factors. Nucleic Acids Res..

[67] Seemann T. Snippy: Fast Bacterial Variant Calling from NGS Reads, 2022. https://github.com/tseemann/snippy.

[68] Seemann T. Snp-Dists: Pairwise SNP Distance Matrix from a FASTA Sequence Alignment 2022. https://github.com/tseemann/snp-dists.

[69] Letunic I., Bork P. (2007). Interactive Tree of Life (ITOL): An Online Tool for Phylogenetic Tree Display and Annotation. Bioinforma. Oxf. Engl..

[70] Wickham H. (2016). Ggplot2: Elegant Graphics for Data Analysis.

[71] Wickham H., François R., Henry L., Müller K. (2021). Dplyr: A Grammar of Data Manipulation. R Package Version 1.0.5. https://dplyr.tidyverse.org.

[72] Wickham H. (2019). Stringr: Simple, Consistent Wrappers for Common String Operations. R Package Version 1.4.0. https://stringr.tidyverse.org.

[73] Wickham H. (2020). Tidyr: Tidy Messy Data. R Package Version 1.1.2. https://github.com/tidyverse/tidyr.

[74] Xie Y. Knitr: A General-Purpose Package for Dynamic Report Generation in R. R Package Version 1.30. https://yihui.org/knitr/.

[75] Zhu H. (2020). KableExtra: Construct Complex Table with “kable” and Pipe Syntax. R Package Version 1.3.1. https://haozhu233.github.io/kableExtra/.

[76] Kaplan J. (2020). FastDummies: Fast Creation of Dummy (Binary) Columns and Rows from Categorical Variables. R Package Version 1.6.3. https://github.com/jacobkap/fastDummies.

