# Supplementary material for: Clonal Lineages and Virulence Factors of Carbapenem Resistant E. coli in Alameda County, California, 2017–2019
Source: Antibiotics (Basel). 2022 Dec 10;11(12):1794. doi: 10.3390/antibiotics11121794 (PMC9774732; doi:10.3390/antibiotics11121794)
Supplement: Supplementary file 1 [file antibiotics-11-01794-s001.zip › antibiotics-1913001-supplementary.pdf]

Supplement File S1.

Table S1. Summary of antibiotic resistance gene frequencies in CRE isolates.

Antibiotic Class	Resistance Genes Identified	Isolates Containing Resistance Genes (n=82)	Isolates Containing Antibiotic Class (%)
B-Lactams	*bla* CMY-138, *bla* CMY-140, *bla* CMY-145,	1/82, 1/82, 4/82,	74/82 (90.2)
	bla CMY-2, *bla* CMY-42, *bla* CMY-6	7/82, 8/82, 1/82	
	bla CTX_M-14, *bla* CTX_M-15, *bla* CTX_M-27,	5/82, 29/82, 5/82,	
	bla CTX_M-3, *bla* CTX_M-55, *bla* CTX_M-65	1/82, 5/82, 2/82	
	bla SHV-12	1/82	
	bla TEM-1B, *bla* TEM-1C, *bla* TEM-30, *bla* TEM-141	28/82, 1/82, 1/82, 1/82	
	bla OXA-1	25/82	
Aminoglycosides	*Aac(3)-Iia_1, aac(3)-Iid_1,*	10/82, 8/82,	65/82 (79.3)
	aac(3)-VIa_2, aac(6')-Ib-cr_1	1/82, 25/82	
	aadA2_1, aadA5_1, ant(3'')-Ia_1,	19/82, 27/82, 4/82,	
	ant(3')-Ia_1, aph(3'')-Ib_2,	3/82, 1/82,	
	aph(3'')-Ib_5, aph(6)-Id_1	30/82, 33/82	
	armA_1	1/82	
	rmtB_1	3/82	
Tetracyclines	*tet*(A)_6, *tet*(B)_2, *tet*(W)_5	28/82, 15/82 , 1/82	43/82 (52.4)
Sulfonamides	*sul*1_5, *sul*2_2, *sul*2_3, *sul*2_6, *sul*3_2	43/82, 27/82, 4/82, 1/82, 1/82	56/82 (68.3)
Fluoroquinolones	*qep*A4_1, *qnr*B1_1, *qnr*S1_1	1/82, 3/82, 8/82	11/82 (13.4)
Trimethoprim	*dfr*A1_10, *dfr*A12_8, *dfr*A14_5,	1/82, 19/82, 10/82,	51/82 (62.2)
	*dfr*A17_1, dfrA5_1, *dfr*A8_1	26/82, 1/82, 1/82	
Macrolides	*erm*(B)_1, *erm*(B)_18, *mef*(C)_1,	2/82, 7/82, 1/82,	38/82 (46.3)
	mph(A)_2, *mph*(B)_1, *mph*(E)_1,	32/82, 1/82, 2/82,	
	mph(G)_1, *msr*(E)_1	1/82, 2/82	
Chloramphenicols	*cat*A1_1, *cat*B3_2, *flo*R_2	2/82, 3/82, 6/82	11/82 (13.4)
Multi-Drug Resistant	*mdf*(A)_1	82/82	82/82 (100%)

Table S2. Summary of isolate collection meta-data from Alameda County, California, 2017-2019

Collection Category	Specimen Source (n)	Healthcare Setting (n)	ST (n=82)
Blood	Blood (6)	Hospital (6)	131 ,405, 69, 6870, 73 , UNK
Body fluids- Other	Sputum (3)	Hospital (3)	131, 648, UNK
	Peritoneal Fluid	Hospital	6870
	Dialyste Fluid	Hospital	6870
Enteric	Stool (2)	Hospital (2)	122, 11
	Rectal Swab (7)	LTAC (7)	131(2), 405(2), 410, 457,UNK
	Rectal Swab (4)	Hospital (4)	69, 167, 10, 156
Other	Bile	Hospital	44
	Right Kidney	Hospital	90
Tissue	Abscess (3)	Hospital(3)	131(2), 10
	Jp drainage	Hospital	101
	Sacral tissue	Hospital	405
Urine	Urine (47)	Hospital (47)	131(10), 405(8), 410(3), 90(3), 345(3),
			354(2), 648(3), 38(3), 69(2), 167(2),
			156 ,144, 167, 963, 1193, 10, UNK(2)
Unknown	Unknown	Hospital (4)	131, 95, 167, 404

*LTAC – long-term acute care facility

Figure S1. Pearson correlation coefficients of isolate collection meta-data from Alameda County, California,

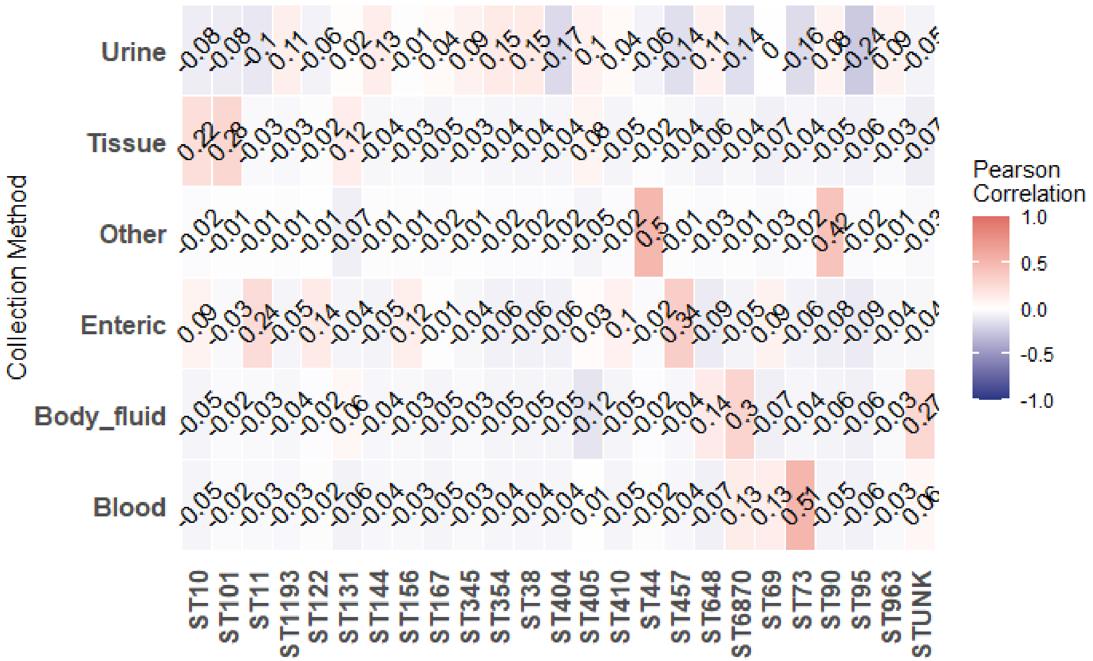

2017-2019

Table S3. Isolate meta data, ST type, and carbapenemase resistance genes from isolates collected from the same patients from Alameda County, California, 2017–2019

Patient	Isolate	Collection Category	Specimen Source (n)	Healthcare Setting (n)	ST	Carbapenemase Resistance Genes
A	ID1000301	Body Fluid	Peritoneal Fluid	Hospital	6870	*bla*NDM-5
	ID1000892	Body Fluid	Dialysate Fluid	Hospital	6870	*bla*NDM-5
	ID1000918	Blood	Blood	Hospital	6870	*bla*NDM-5
B	ID1000213	Urine	Urine	Hospital	90	*bla*NDM-1
	ID1000641	Urine	Urine	hospital	90	None
	ID1000771	Urine	Urine	hospital	90	None
	ID1000873	Other	Right Kidney	hospital	90	*bla*NDM-1
C	ID1000152	Urine	Urine	Hospital	167	*bla*NDM-5
	ID1000835	Urine	Urine	Hospital	167	*bla*NDM-5
D	ID1000627	Urine	Urine	Hospital	354	None
	ID1000893	Urine	Urine	hospital	354	None
E	ID1000247	Urine	Urine	Hospital	405	None
	ID1000739	Urine	Urine	hospital	405	None
F	ID1000270	Enteric	Rectal Swab	LTAC	405	None
	ID1000492	Enteric	Rectal Swab	LTAC	405	None
G	ID1000461	Urine	Urine	Hospital	345	None
	ID1000399	Urine	Urine	Hospital	345	None
	ID1000095	Urine	Urine	Hospital	345	None
